# Predictors of Test Positivity, Mortality, and Seropositivity during the Early Coronavirus Disease Epidemic, Orange County, California, USA

**DOI:** 10.3201/eid2710.210103

**Published:** 2021-10

**Authors:** Daniel M. Parker, Tim Bruckner, Verónica M. Vieira, Catalina Medina, Vladimir N. Minin, Philip L. Felgner, Alissa Dratch, Matthew Zahn, Scott M. Bartell, Bernadette Boden-Albala

**Affiliations:** University of California, Irvine, Irvine, California, USA (D.M. Parker, T. Bruckner, V.M. Vieira, C. Medina, V.N. Minin, P.L. Felgner, S.M. Bartell, B. Boden-Albala);; Orange County Health Care Agency, Santa Ana, California, USA (A. Dratch, M. Zahn)

**Keywords:** COVID-19, coronavirus disease, SARS-CoV-2, severe acute respiratory syndrome coronavirus 2, viruses, respiratory infections, zoonoses, test positivity, mortality, seropositivity, California, Orange County, United States, health equity

## Abstract

We conducted a detailed analysis of coronavirus disease in a large population center in southern California, USA (Orange County, population 3.2 million), to determine heterogeneity in risks for infection, test positivity, and death. We used a combination of datasets, including a population-representative seroprevalence survey, to assess the actual burden of disease and testing intensity, test positivity, and mortality. In the first month of the local epidemic (March 2020), case incidence clustered in high-income areas. This pattern quickly shifted, and cases next clustered in much higher rates in the north-central area of the county, which has a lower socioeconomic status. Beginning in April 2020, a concentration of reported cases, test positivity, testing intensity, and seropositivity in a north-central area persisted. At the individual level, several factors (e.g., age, race or ethnicity, and ZIP codes with low educational attainment) strongly affected risk for seropositivity and death.

In late 2019, an epidemic of coronavirus disease (COVID-19), a respiratory disease caused by a novel coronavirus, severe acute respiratory syndrome coronavirus 2 (SARS-CoV-2), emerged in Wuhan, China, and rapidly spread worldwide. COVID-19 has manifested in different ways across social, economic, and demographic groups, with regard to apparent risk for infection, disease severity, and death (*1*–*3*). The elderly and those with underlying conditions are at the highest risk for severe disease (*4*). Many hospitalized patients require supplemental oxygen or ventilators (*5*), and a high mortality rate occurs among those who are hospitalized (*6*). In many places, healthcare facilities have been overwhelmed by a surge in cases and have had an insufficient supply of needed ventilators and intensive care unit beds, resulting in massive illness and death (*7*,*8*). Availability of tests and operational barriers were limiting factors for diagnosis in parts of the United States during the early months of the pandemic (*9*).

California is the most populous state in the United States, and it was an estimated 39.5 million inhabitants. Orange County (OC) is a coastal county in California and the sixth most populous county in the country, with an estimated 3.2 million inhabitants. The first confirmed case in California (the third US case) was reported from OC on January 25 (*10*). On January 30, the World Health Organization declared a global health emergency (*11*), and on January 31, the United States likewise declared a public health emergency (*12*). On February 26, local (i.e., community) transmission was first confirmed in the United States in northern California (*13*). Several counties by this time had already declared local public health emergencies, including Santa Clara (*14*), San Diego (*15*), and Orange (*16*) Counties, as had the city of San Francisco (*17*). By mid-March, an apparent surge in locally transmitted cases was occurring in OC (Figure 1) and other counties in California, triggering emergency shelter-in-place orders by the governor and the county health officer at the Orange County Health Care Agency (OCHCA), prohibiting public or private gatherings and also leading to school and business closures (*18*). Although many businesses were closed at this time, the mandated social distancing measures had exceptions in place for persons working in essential jobs, which was broadly defined and included medical professionals, food providers, delivery agencies, public officials, construction contractors, and building laborers (*19*). The social and economic characteristics of persons working essential jobs differs from the overall population (*20*).

**Figure 1:**
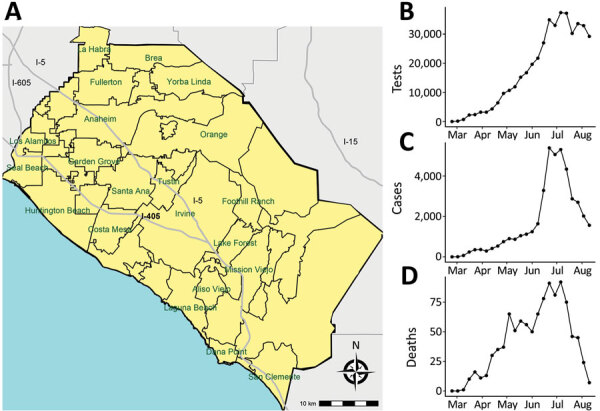
Locations of major cities (A), number of weekly severe acute respiratory syndrome coronavirus 2 tests (B), weekly confirmed coronavirus disease cases (C), and weekly coronavirus disease deaths (D), Orange County, California, USA, July–August 2020.

Almost half of OC residents >5 years of age speak a language other than English at home. In addition, many within the Hispanic/Latinx and Asian communities of OC live below the poverty level (17.9% and 12.0%, respectively) and face challenges in education, household income, access to healthcare, health disparities, and life expectancy (*21*,*22*). The relatively small land area, high population density, and diverse population of OC provides a unique opportunity to explore potentially important social, economic, and demographic correlates of COVID-19 epidemiology.

We conducted a detailed spatiotemporal epidemiologic analysis of COVID-19 in OC during March 1–August 16, 2020. We drew from reported tests and mortality data from the county health agency. Given that passively detected cases are prone toward bias, in July 2020 we also conducted a seroprevalence survey to assess the actual burden of disease in the county. We use both datasets to compare predictors of test positivity, death, and seropositivity over the first 6 months of the epidemic.

## Methods

### Data

#### Case and Mortality Data

Case data were provided by OCHCA and consisted of individual-level records of all negative and positive PCR tests conducted throughout the county during March 1–August 16, 2020; this date aligns with our cross-sectional seroprevalence survey completed on August 16. OCHCA receives testing data from the California Reportable Disease Information Exchange (CalREDIE), an infectious disease surveillance system implemented by the California Department of Public Health (*14*). The data include test date, age, sex, race, ethnicity, and ZIP code of the person taking the test. For persons who had repeat PCR testing after testing positive, we included only the first positive diagnosis in our analyses; we retained multiple negative test results. Mortality data were also provided by OCHCA (also through CalREDIE) and consisted of individual-level records of deaths attributed to COVID-19. OCHCA likewise provided data on the number and percentage of hospital beds that were occupied by COVID-19 patients over time.

#### Seroprevalence Data

Participants in the serologic survey were recruited using a proprietary database (Appendix), which is intended to be representative of the age, income, and racial and ethnic diversity of OC. We recruited 1 participant per household (by email or phone) to participate in a survey on their thoughts and opinions regarding COVID-19. The survey included questions on sociodemographics, occupation, social activities, any illness or symptoms in the past few months, and whether the person had been diagnosed with COVID-19. After completing this portion of the survey, each eligible participant was asked if they would be willing to participate in a drive-through blood test for SARS-CoV-2 antibodies. Eligibility for antibody testing was restricted to a quota sample designed to be demographically representative of the county as a whole. Recruitment to the antibody test was delayed to the end of the questionnaire to avoid biasing the serologic survey toward persons who believed that they were infected with SARS-CoV-2. A total of 10 field sites for drive-through blood tests were dispersed throughout OC to minimize driving distances for participants. This cross-sectional survey was conducted July 10–August 16, 2020. The seroprevalence study design and overall findings for OC have been described previously (*23*).

#### Serologic Test Data

We used a coronavirus antigen microarray to classify participants from the serologic survey as seropositive or seronegative. The array tests for IgG and IgM and contains 12 antigens from SARS-CoV-2 (R.R. de Assis et al., unpub. data, https://doi.org/10.1101/2020.04.15.043364).

#### ZIP Code–Level Sociodemographic Data

At the ZIP code level, we included median household income, the percentage of adults >25 years of age with at least a bachelor’s degree, the percentage of adults who had health insurance in the previous 5 years, the number of persons per square kilometer, and the percentage of houses with >1 person per room. These data came from the 2018 American Community Survey (*21*).

### Analysis

#### Descriptive Spatiotemporal Data Analysis

We aggregated reported cases and number of tests at the ZIP code level and by week. We included 86 ZIP codes in the analysis. For plotting cases on OC maps, we further aggregated the data by month (March–August). We calculated and mapped case incidence as positive cases per 100,000 population per week, testing intensity as total number of tests per 100,000 persons per week, and test positivity as the percentage of positive tests for each month.

We conducted formal testing of spatial autocorrelation by using the global Moran’s *I* statistic and spatial correlograms. We then used local clustering statistics (local indicators of spatial autocorrelation [LISA] [*24*]) to visualize the location of clusters. We ran all tests for case incidence, test positivity, and test intensity. We also used LISA statistics to map and assess seropositivity. 

#### Relational Analysis of COVID-19 Test Positivity, Risk for Death, and Seropositivity

We used logistic regressions to explore geographic, demographic, economic, and epidemiologic predictors of the odds of testing positive for COVID-19, of dying from COVID-19, and of being seropositive for SARS-CoV-2 antibodies. Predictors in our models were age group, sex, and race or ethnicity at the individual level (Appendix Table 1). ZIP code–level predictors were median household income, the percentage of adults >25 years of age with at least a bachelor’s degree, the percentage of adults who had health insurance in the previous 5 years, population density (persons/km^2^), and house crowding (the percentage of houses with >1 person per room).

We tested several specifications of the models. Through preliminary exploratory analyses, we noted that the first cases were reported from coastal ZIP codes but that this pattern had shifted inland over time. The best fitting model included a smoothed interaction term for time, coded by day (Appendix Table 1), and median household income at the ZIP code level.

We included the same predictors in the model for risk for death, except for the interaction between time and median household income, which did not improve model performance. Given reports of increased mortality rates related to hospital bed shortages, we also included as a predictor the number of intensive care unit beds occupied by suspected or confirmed COVID-19 patients on the day that any person tested positive for SARS-CoV-2. For all model results, we calculated model-adjusted odds ratios (aORs) with 95% CIs.

#### Software

We created maps by using QGIS 3.4.9 (https://qgis.org). We conducted tests for spatial autocorrelation by using GeoDa 1.14.0 (https://geodacenter.github.io) and all other analyses by using R statistical software 3.5.2 (R Project for Statistical Computing, https://www.r-project.org).

#### Ethics Considerations

This analysis constitutes a retrospective analysis of deidentified, anonymized epidemiologic records. Therefore, it is exempt from ethics review. 

## Results

A total of 597,922 tests were reported to OCHCA through August 16, 2020. After excluding repeated tests and those with incomplete data, 316,626 (53.0% of all records) persons were included in the test positivity analysis; 37,546 (12.0%) persons tested positive for COVID-19. A total of 42,383 persons with positive COVID-19 tests were included in the mortality analysis; 1,038 (2.5%) died from the disease. In the separate population-based serologic survey, 2,979 persons participated and 350 tested seropositive.

### Spatial Patterns in Reported COVID-19 Cases, Testing Intensity, and Seropositivity

**Figure 2:**
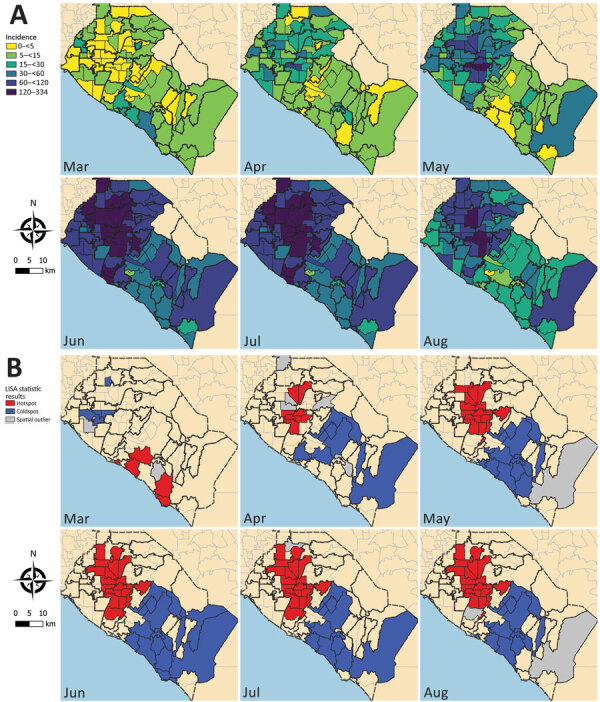
Coronavirus disease incidence, Orange County, California, USA, July–August 2020. A) Reported case incidence of coronavirus by month. Case incidence is calculated as the number of cases per 100,000 persons per week.B) Results from tests of statistical clustering (based on LISA statistics [*24*]). LISA, local indicators of spatial autocorrelation.

**Figure 3:**
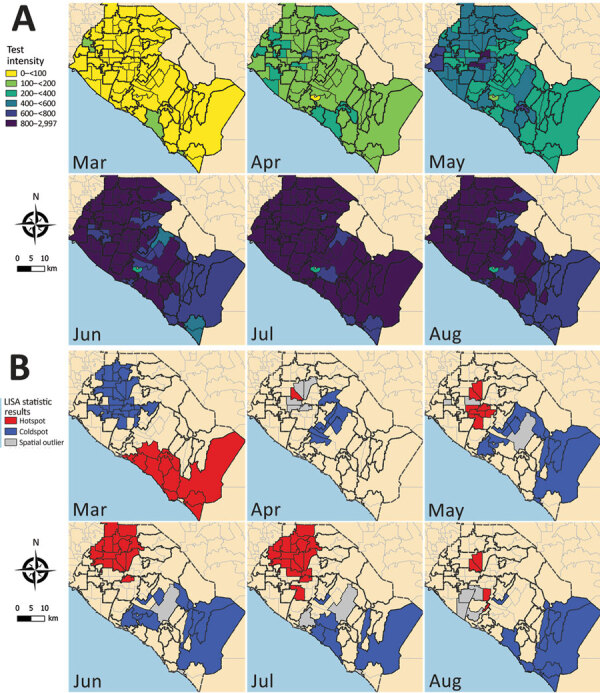
Severe acute respiratory syndrome coronavirus 2 test intensity, Orange County, California, USA, July–August 2020. A) Test intensity by month, calculated as the number of tests per 100,000 persons per week at the ZIP code level. B) Results from tests of statistical clustering (based on LISA statistics [*24*]). LISA, local indicators of spatial autocorrelation.

The tests for spatial autocorrelation indicated significant clustering in reported cases and testing intensity in the first month (March) of the local epidemic (Table 1; Appendix Figures 1, 2). Conversely, no detectable clustering of test positivity occurred in March (Table 1; Appendix Figure 3). The highest reported case incidence in March was along the central coast and southern portion of the county (Figure 2, panel A). The LISA statistics indicated statistically significant clustering of high-incidence ZIP codes in the central coast area (Figure 2, panel B). This clustering of case incidence overlaps with clustering of test intensity in March (Figure 3, panels A, B).

**Table 1:** Global Moran’s *I* statistics for reported coronavirus disease case incidence, test positivity, and testing intensity for each month of the study period, Orange County, California, USA, March–August 2020*

Month	Case incidence			Test positivity			Testing intensity	
	*I*	p value		*I*	p value		*I*	p value
March	0.238	0.002		0.059	0.150		0.448	0.001
April	0.168	0.012		0.271	0.001		0.022	0.257
May	0.558	0.001		0.492	0.001		0.345	0.001
June	0.606	0.001		0.552	0.001		0.469	0.001
July	0.591	0.001		0.500	0.001		0.408	0.001
August	0.603	0.001		0.472	0.001		0.185	0.002

*The Moran’s *I* statistic indicates the degree of spatial clustering whereas the simulated p-value gives an indication of statistical significance. Moran’s *I* values roughly range from −1 to 1, with 1 indicating complete spatial clustering (i.e., all areas with high values are neighboring other areas with high values) and −1 indicating complete spatial dispersion (with high value areas always neighboring low value areas).

**Figure 4:**
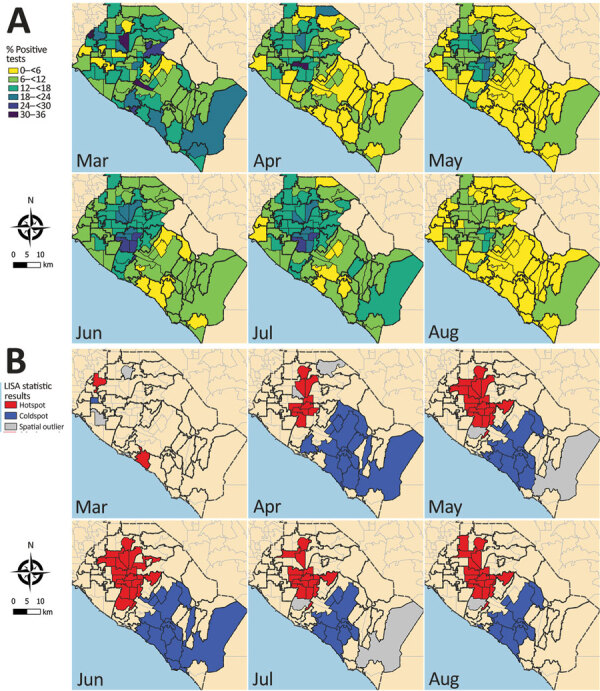
Severe acute respiratory syndrome coronavirus 2 test positivity, Orange County, California, USA, July–August 2020. A) Test positivity at ZIP code level by month. B) Results from tests of statistical clustering (based on LISA statistics [*24*]). LISA, local indicators of spatial autocorrelation.

**Figure 5:**
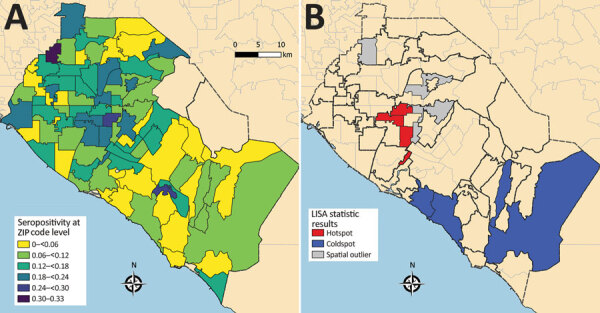
Severe acute respiratory syndrome coronavirus 2 seropositivity, Orange County, California, USA, July–August 2020. A) Seropositivity at ZIP code level. B) Results from tests of statistical clustering (based on LISA statistics [*24*]). LISA, local indicators of spatial autocorrelation.

Clustering of reported cases and test positivity increased in magnitude in May (Table 1; Appendix Figures 1, 3). Although clustering in test intensity was high in March (Table 1; Appendix Figure 2), it decreased in May as access to testing spread throughout much of the county. Clustering in testing intensity increased again in June and July (centered on the hotspots in the north-central part of the county) (Figure 2, 4). By April, case incidence, testing intensity, and test positivity had all shifted to the north-central part of the county. ZIP code–level seropositivity also revealed a cluster in the north-central part of OC (Figure 5), especially in the city of Santa Ana (Figure 1).

### Results from Generalized Additive Model Logistic Regression Analysis

#### Factors Associated with Testing Positive for SARS-CoV-2 infection

**Figure 6:**
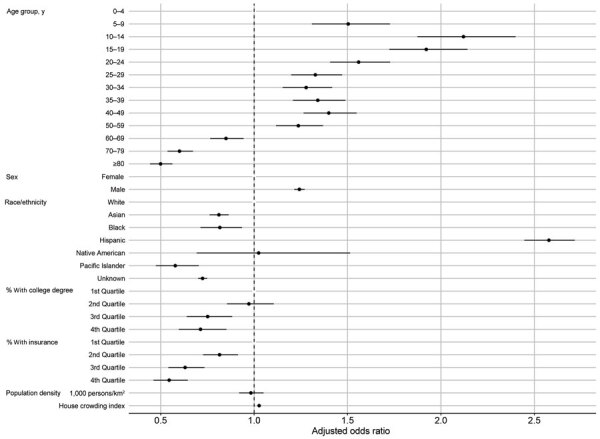
Model-adjusted odds ratios and 95% CIs from the logistic regression for odds of testing positive for severe acute respiratory syndrome coronavirus 2, Orange County, California, USA, July–August 2020. Corresponding data presented in Table 2.

Age was a strong predictor of testing positive. Persons in the 10–14- and 15–19-year age groups had the highest odds of testing positive (both with ≈2.30 times the odds of testing positive compared with the 0–4 year age group) (Table 2; Figure 6). Men and boys had 1.20 times the odds of testing positive than women and girls (95% 95% CI 1.18–1.23). Persons who identified as Hispanic or Latinx had 1.7 times the odds of testing positive (95% CI 1.60–1.76) than did non-Hispanic Whites, whereas Asian (aOR 0.55; 95% CI 0.52–0.58), Black (aOR 0.58; 95% CI 0.51–0.65), and Pacific Islander (aOR 0.35; 95% CI 0.29–0.42) persons had lower odds of testing positive than did non-Hispanic Whites. A large proportion of persons did not have attributable race or ethnicity data in the records (72% of all records through August 16). This unknown category includes persons who had no race or ethnicity categories recorded, those who had unknown or mixed listed for race or ethnicity, and those who listed multiple races.

**Table 2:** Generalized additive logistic regression results for odds of testing positive for SARS-CoV-2, Orange County, California, USA, March–August 2020*

Characteristic	No. (%)		Adjusted odds ratio† (95% CI)
	SARS-CoV-2 positive	Total tests	
Age group, y			
0–4	487 (1.3)	4,835 (1.53)	Referent
5–9	490 (1.31)	3,855 (1.22)	1.62 (1.41–1.86)
10–14	855 (2.28)	5,064 (1.6)	2.26 (2.00–2.56)
15–19	2,124 (5.66)	13,814 (4.36)	2.32 (2.08–2.58)
20–24	4,646 (12.37)	31,727 (10.02)	2.04 (1.85–2.26)
25–29	4,640 (12.36)	34,695 (10.96)	1.74 (1.57–1.93)
30–34	3,791 (10.1)	29,900 (9.44)	1.62 (1.46–1.79)
35–39	3,291 (8.77)	25,776 (8.14)	1.67 (1.5–1.85)
40–49	5,950 (15.85)	44,835 (14.16)	1.75 (1.58–1.93)
50–59	5,747 (15.31)	48,502 (15.32)	1.54 (1.39–1.71)
60–69	3,045 (8.11)	36,294 (11.46)	1.04 (0.94–1.16)
70–79	1,404 (3.74)	22,190 (7.01)	0.77 (0.69–0.86)
>80	1,076 (2.87)	15,139 (4.78)	0.80 (0.72–0.9)
Sex			
F	19,076 (50.81)	173,723 (54.87)	Referent
M	18,470 (49.19)	142,903 (45.13)	1.20 (1.18–1.23)
Race or ethnicity			
White	12,195 (32.48)	63,050 (19.91)	Referent
Asian	1,573 (4.19)	13,858 (4.38)	0.55 (0.52–0.58)
Black	289 (0.77)	2,058 (0.65)	0.58 (0.51–0.65)
Hispanic	3,473 (9.25)	9,147 (2.89)	1.68 (1.6–1.76)
Native American	56 (0.15)	314 (0.1)	0.82 (0.62–1.09)
Pacific Islander	127 (0.34)	1,600 (0.51)	0.35 (0.29–0.42)
Unknown	19,833 (52.82)	226,599 (71.57)	0.32 (0.31–0.33)
% Persons with college degree in ZIP code			
1st quartile	20,665 (55.04)	120,279 (37.99)	Referent
2nd quartile	9,484 (25.26)	87,802 (27.73)	0.89 (0.77–1.03)
3rd quartile	4,560 (12.15)	64,604 (20.4)	0.70 (0.58–0.84)
4th quartile	2,837 (7.56)	43,941 (13.88)	0.68 (0.56–0.83)
% Persons with insurance in ZIP code			
1st quartile	19,749 (52.6)	111,798 (35.31)	Referent
2nd quartile	10,371 (27.62)	93,431 (29.51)	0.83 (0.73–0.95)
3rd quartile	3,824 (10.18)	53,201 (16.8)	0.67 (0.56–0.8)
4th quartile	3,602 (9.59)	58,196 (18.38)	0.58 (0.48–0.7)
Population density, × 1,000 persons/km^2^‡			0.97 (0.9–1.04)
House crowding			1.03 (1.02–1.04)

*Excludes the coefficients for ZIP code–level median income and time because of interaction between median income and time. A random intercept was included for ZIP code. The period covered in this analysis is March 1–August 16, 2020. SARS-CoV-2, severe acute respiratory syndrome coronavirus 2. †Adjusted for all covariates listed plus ZIP code estimated median income and time of test in days. Model intercept represents odds of a White female in the 0–4-y age group in a ZIP code in the first quartile of college degree and insured with the average population density. The odds of this person testing positive for SARS-CoV-2 is estimated to be 0.19 (95% CI 0.16–0.22). ‡Estimated percentage of population density in a person’s ZIP code.

**Figure 7:**
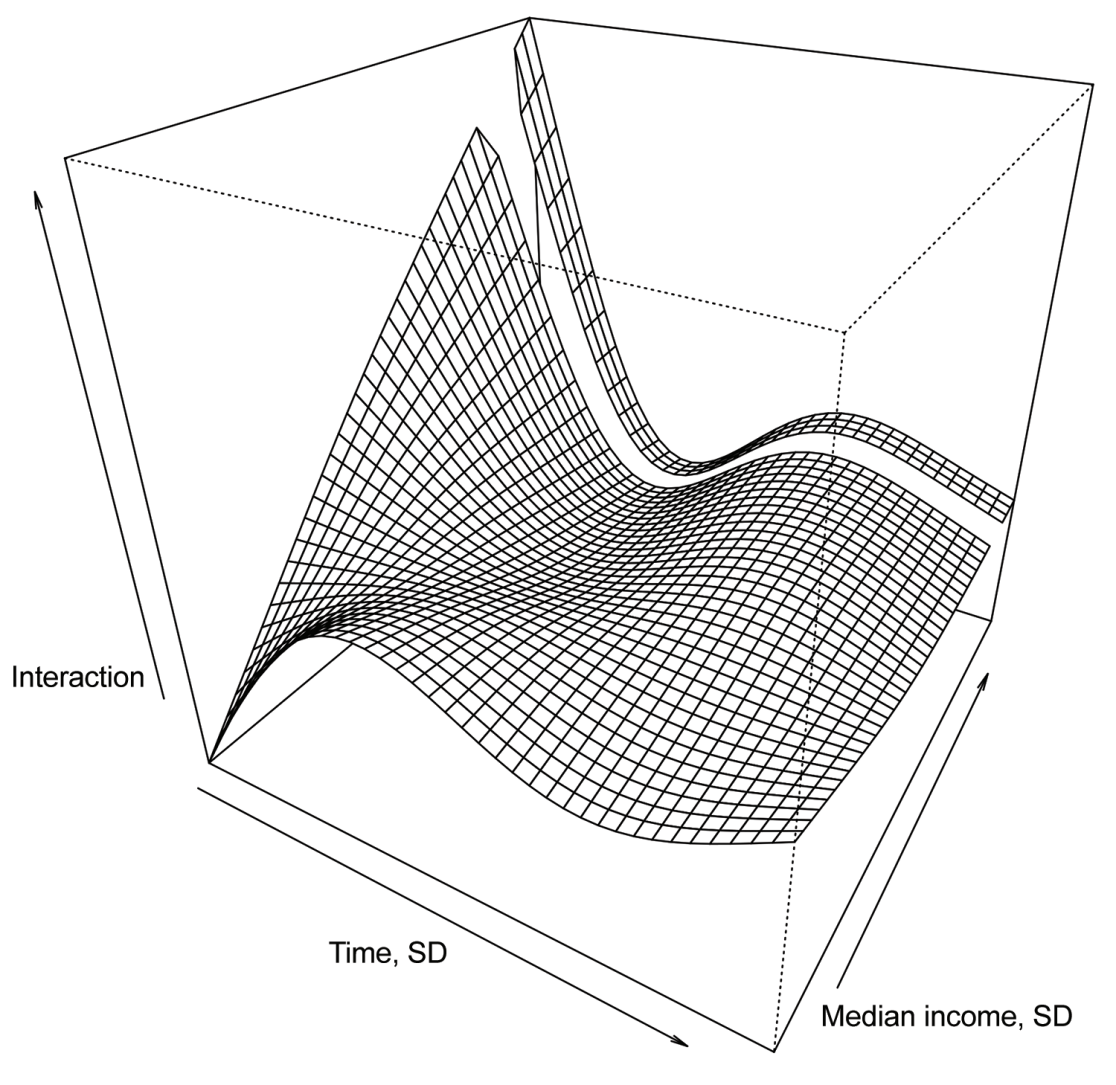
Three dimensional plot of the smoothed interaction between ZIP code–level median household income and time as a predictor of testing positive for severe acute respiratory syndrome coronavirus 2, Orange County, California, USA, July–August 2020.

ZIP code–level population density was not a significant predictor of testing positive (Table 2; Figure 6). However, education (percentage of adults >25 years of age with at least a bachelor’s degree), health insurance coverage (percentage of adults who had health insurance in the previous 5 years), median household income, and household crowding were all statistically significant predictors of testing positive. For example, persons who lived in ZIP codes with the highest education levels had 32% decreased odds of testing positive (aOR for the fourth quartile 0.68, 95% CI 0.56–0.83). In addition, the interaction between ZIP code–level median household income (Figure 7) indicates that persons from wealthier ZIP codes had increased risk for testing positive at the beginning of the epidemic in OC. However, this pattern quickly shifted, and persons from lower-income areas showed the highest odds of testing positive in subsequent months.

#### Factors Associated with Dying from COVID-19

**Figure 8:**
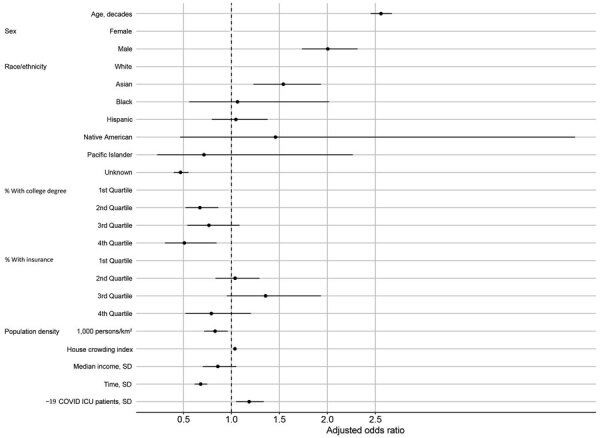
Model-adjusted odds ratios and 95% CIs from the logistic regression for the odds of dying from COVID-19, Orange County, California, USA, July–August 2020. Corresponding data presented in Table 3. COVID-19, coronavirus disease; ICU, intensive care unit.

For each increase in 10 years of age, we observed an associated 2.5-fold increase in the odds of death (aOR 2.56, 95% CI 2.45–2.67; Table 3; Figure 8). Infected men and boys were twice as likely to die from COVID-19 than were women and girls (aOR 2.00, 95% CI 1.73–2.31). Although persons who identified as Asian were less likely to test positive for SARS-CoV-2 infection (Table 2), those who did test positive had higher odds of death. Compared with non-Hispanic Whites, this group had 54% increased odds of dying from COVID-19 (aOR 1.54, 95% CI 1.23–1.93).

**Table 3:** Logistic regression results for odds of dying from COVID-19 among persons who tested positive for SARS-CoV-2, Orange County, California, USA, March–August 2020*

Characteristic	No. (%)		Adjusted odds ratio (95% CI)†
	COVID-19 deaths, n = 1,038	Total cases, n = 42,383	
Age, decades			2.56 (2.45–2.67)
Sex			
F	450 (43.35)	21,694 (51.19)	Referent
M	588 (56.65)	20,689 (48.81)	2.00 (1.73–2.31)
Race or ethnicity			
White	345 (33.24)	6,390 (15.08)	Referent
Asian	186 (17.92)	1,963 (4.63)	1.54 (1.23–1.93)
Black	15 (1.45)	322 (0.76)	1.06 (0.56–2.02)
Hispanic	92 (8.86)	3,874 (9.14)	1.05 (0.79–1.38)
Native American	3 (0.29)	34 (0.08)	1.46 (0.46–4.58)
Pacific Islander	3 (0.29)	130 (0.31)	0.71 (0.22–2.26)
Unknown	394 (37.96)	29,670 (70)	0.47 (0.4–0.55)
% With college degree in ZIP code			
1st quartile	656 (63.2)	23,221 (54.79)	Referent
2nd quartile	190 (18.3)	10,223 (24.12)	0.67 (0.52–0.86)
3rd quartile	155 (14.93)	5,691 (13.43)	0.77 (0.54–1.08)
4th quartile	37 (3.56)	3,248 (7.66)	0.51 (0.31–0.84)
% With insurance in ZIP code			
1st quartile	566 (54.53)	21,989 (51.88)	Referent
2nd quartile	281 (27.07)	11,097 (26.18)	1.04 (0.83–1.29)
3rd quartile	123 (11.85)	5,185 (12.23)	1.36 (0.95–1.93)
4th quartile	68 (6.55)	4,112 (9.7)	0.79 (0.52–1.2)
Population density, × 1,000 persons/km^2^‡			0.83 (0.71–0.96)
House crowding index			1.04 (1.02–1.05)
Median income (SD)			0.86 (0.7–1.05)
Time (SD)			0.68 (0.62–0.75)
COVID-19 ICU patients (SD)§			1.18 (1.05–1.34)

*Values are no. (%) except where indicated. A random intercept was included for ZIP code. The period covered in this analysis is March 1–August 16, 2020. Total numbers of positive cases are larger than the total number reported in Table 2 because of more extensive data curation for mortality data than for general test data. More rows of data were dropped because of missing information (e.g., on age or sex) in the test positivity data than in the mortality data. COVID-19, coronavirus disease; ICU, intensive care unit; SARS-CoV-2, severe acute respiratory syndrome coronavirus 2. †Model intercept represents odds of death for a White female diagnosed with SARS-CoV-2 in the 0–4 years age group in a ZIP code in the first quartile of college degree and insured with the average population density in Orange County. The odds of this person testing dying from COVID-19 is estimated to be zero. ‡Estimated percentage of population density in a person’s ZIP code. §Percentage of hospital beds not being used by COVID-19 patients in Orange County.

Living in ZIP codes with high education levels and health insurance coverage was also predictive of mortality outcomes (Table 3; Figure 8). Persons who tested positive for COVID-19 and lived in ZIP codes with the highest levels of educational attainment had 49% lower odds of dying from COVID-19 (aOR for the fourth quartile 0.51, 95% CI 0.31–0.84). Persons who lived in ZIP codes with the highest levels of health insurance coverage had 21% lower odds of dying from COVID-19. ZIP code–level household crowding and the number of COVID-19 patients in hospital beds were both significant predictors of death. Risk for death from COVID-19 decreased over the study period.

#### Factors Associated with SARS-CoV-2 Seropositivity

ZIP code–level cumulative incidence was a significant predictor of individual-level seropositivity in the absence of other ZIP code–level predictors. Every increase in 10% of the ZIP code cumulative incidence resulted in an approximately 50% increase in the odds that a person would be seropositive (Appendix Table 2).

**Figure 9:**
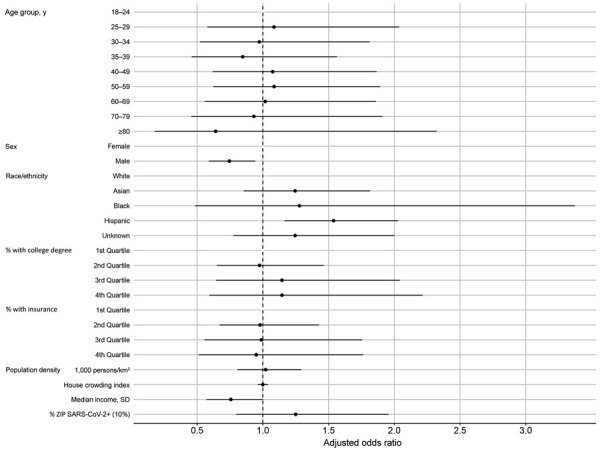
Model-adjusted odds ratios and 95% CIs from the logistic regression for the odds of being seropositive for severe acute respiratory syndrome coronavirus 2, Orange County, California, USA, July–August 2020. Corresponding data presented in Table 4.. +, positive.

ZIP code–level cumulative incidence was no longer a statistically significant predictor of seropositivity when other ZIP code–level predictors were added to the model (Table 4; Figure 9). In the full model (including all ZIP code–level covariates), median household income had a protective effect; persons coming from ZIP codes with higher median household income had lower odds of being seropositive for SARS-CoV-2 antibodies (aOR for every 1 SD increase 0.75, 95% CI 0.57–1.00).

**Table 4:** Logistic regression results for odds ratio of testing seropositive for SARS-CoV-2, Orange County, California, USA, July–August 2020*

Characteristic	No. (%)		Adjusted odds ratio (95% CI)†
	SARS-CoV-2 seropositive, n = 350	Total tested, n = 2,604	
Age group, y			
18–24	19 (5.43)	158 (5.35)	Referent
25–29	31 (8.86)	234 (7.92)	1.09 (0.58–2.04)
30–34	33 (9.43)	275 (9.31)	0.97 (0.52–1.81)
35–39	35 (10)	328 (11.1)	0.85 (0.46–1.56)
40–49	83 (23.71)	651 (22.04)	1.08 (0.62–1.87)
50–59	82 (23.43)	659 (22.31)	1.09 (0.62–1.89)
60–69	46 (13.14)	418 (14.15)	1.02 (0.56–1.86)
70–79	18 (5.14)	188 (6.36)	0.93 (0.45–1.91)
>80	3 (0.86)	43 (1.46)	0.64 (0.18–2.32)
Sex			
F	222 (63.43)	1,668 (56.47)	Referent
M	128 (36.57)	1,286 (43.53)	0.75 (0.59–0.94)
Race or ethnicity‡			
White	108 (30.86)	1,228 (41.57)	Referent
Asian	47 (13.43)	435 (14.73)	1.25 (0.85–1.82)
Black	5 (1.43)	42 (1.42)	1.28 (0.48–3.37)
Hispanic	162 (46.29)	1,010 (34.19)	1.54 (1.17–2.03)
Pacific Islander	3 (0.86)	12 (0.41)	3.89 (1.04–14.65)
Unknown	25 (7.14)	227 (7.68)	1.25 (0.78–2)
% With college degree in ZIP code			
1st quartile	158 (45.14)	937 (31.72)	Referent
2nd quartile	92 (26.29)	893 (30.23)	0.98 (0.65–1.46)
3rd quartile	59 (16.86)	644 (21.8)	1.15 (0.64–2.04)
4th quartile	41 (11.71)	480 (16.25)	1.15 (0.59–2.22)
% With insurance in ZIP code			
1st quartile	154 (44)	928 (31.42)	Referent
2nd quartile	91 (26)	812 (27.49)	0.98 (0.67–1.43)
3rd quartile	54 (15.43)	597 (20.21)	0.99 (0.56–1.76)
4th quartile	51 (14.57)	617 (20.89)	0.95 (0.51–1.76)
Population density, × 1,000 persons/km^2^			1.02 (0.81–1.29)
House crowding index			1.00 (0.96–1.04)
Median income (SD)			0.76 (0.57–1.00)
% Persons in ZIP code SARS-CoV-2 positive >10%§			1.25 (0.80–1.96)

*This cross sectional survey was conducted July 10–August 16, 2020. SARS-CoV-2, severe acute respiratory syndrome coronavirus 2. †Model intercept represents odds of testing seropositive for SARS-CoV-2 for a White female diagnosed with SARS-CoV2 in the 18–24 years age group in a ZIP code in the first quartile of college degree and insured with the average population density, and average percentage of SARS-CoV-2 positive persons in Orange County. The odds of this person testing seropositive is estimated to be 0.074 (95% CI 0.031–0.178). 95% CIs computed with robust SEs. ‡For comparison, the estimated race/ethnicity makeup of Orange County in 2021 is non-Hispanic White (38.6%, n = 1,223,157; Black/African American (1.6%, n = 52,696); American Indian or Alaskan Native (0.3%, n = 6,018); Asian (21.1%, n = 685,728); Native Hawaiian or Pacific Islander (0.3%, n = 8,885); other or multiple races (3.1%, n = 100,297); Hispanic or Latinx (35.0%, n = 1,115,740) (*21*). American Indian or Alaska Native race group not included in analysis because of lack of data; no person of this race group tested seropositive. §Number of persons who tested positive in person’s ZIP code reported to Orange County Public Health Department during March 1–August 16, 2020, divided by estimated population of ZIP code.

We found no difference in age groups with regard to seropositivity. Although men and boys were more likely to test positive or to die from SARS-CoV-2 infection, they were less likely than women and girls to be seropositive (aOR 0.75, 95% CI 0.59–0.94). Hispanic and Latinx persons had 54% increased odds of being seropositive (aOR 1.54, 95% CI 1.17–2.03). Pacific Islanders may also have had higher odds of being seropositive, but with small total numbers and broad 95% CIs (aOR 3.89, 95% CI 1.04–14.65); a total of 3 of 12 Pacific Islanders tested were seropositive.

## Discussion

Infectious disease data from passive case detection can be biased in various ways, including the well-documented challenge of uneven access to testing and diagnosis (*25*) and a general bias toward persons who are seeking clinical care for symptomatic disease. In our analysis of COVID-19 in OC, we used a rich set of complementary data that included those passively collected (e.g., reported cases and mortality records) and those from active screening (e.g., population-based serologic testing). Results indicate that, in the early days of the epidemic in OC, both testing intensity and test positivity were concentrated in wealthy and affluent areas along the central coast. After March, however, a large cluster of reported cases formed in lower-income north-central OC (especially the cities of Santa Ana and Anaheim) (Figures 1, 2), growing in size in May and persisting over time. Testing intensity spread throughout the county during this same period.

Consistent with other reports, we also found that age and male sex strongly predict testing positive and COVID-19 associated death (*26*). Intriguingly, whereas older age groups and men and boys were more likely to have symptomatic disease, our population-based serologic survey found that women and girls were more likely than their male counterparts to be seropositive. Hispanic and Latinx persons had higher risk for infection and testing positive, even after controlling for several ZIP code–level socioeconomic factors. Given the consistency of this finding between the models for test positivity and seropositivity, the risk for being infected with SARS-CoV-2 rises above and beyond the risks of living in a ZIP code with high transmission or a ZIP code with low income and low levels of educational attainment. Other studies also note an increased risk for testing positive among Hispanic and Latinx persons (*27*–*29*). Our seroprevalence survey indicates that in OC, this finding is not an artifact of passive case detection but instead represents an actual true greater risk for infection for Hispanic and Latinx persons.

Although persons identifying as Asian were less likely to test positive for SARS-CoV-2, they were more likely to die when infected. This disparity is consistent with national data, though its cause is uncertain (*30*). This pattern may reflect discrepancies in outreach communication to these communities or other socioeconomic and cultural factors (*31*,*32*) and warrants further detailed investigation.

Social determinants of health, defined as “conditions in which people are born, grow, work, live, age, and the wider set of forces and systems,” play a critical role in the creation of disparities related to illness, death, and quality of life (*33*). These social determinants include (among other factors) poverty, wealth, educational quality, household and neighborhood conditions, childhood experience, and social support. Several speculative explanations have been proposed for these sociodemographic patterns related to COVID-19, including living in dense quarters (and this pattern is evident in our analyses). In addition, as the state and local shelter-in-place and social distancing policies were mandated, persons who are independently wealthy or who work in occupations where working from home was a viable option, were more capable of practicing social distancing. Persons from low socioeconomic status areas, by contrast, may have less ability to practice social distancing. Our analyses show that persons from ZIP codes with lower overall educational attainment and health insurance coverage and with higher housing density were more likely to test positive for and die from COVID-19. The association with median household income was more complex and changed over time with regard to test positivity. However, we also find that persons from ZIP codes with lower median household income were also more likely to be seropositive for SARS-CoV-2. These findings underscore the importance of understanding contextual factors surrounding infectious disease outbreaks.

Study limitations include that county-reported testing and mortality data did not include individual-level information on income, education, and insurance. These variables were only available at the ZIP code–level, and ZIP codes are unlikely to adequately represent important spatial units. Our seroprevalence survey occurred during July 10–August 16, 2020. We limited our analyses of test positivity and risk for death to before August 16, to correspond with the end of the seroprevalence survey. However, the survey occurred over a period of just over a month, during which time the cumulative incidence was changing. Missing data on race and ethnicity (72% of all official test records) and small counts of some racial and ethnic groups may have affected our findings for groups with low counts in this analysis. Even when race or ethnicity data were available, they were broad categories (e.g., Asian rather than specific Asian ethnicities), which is a major limitation of these data, and efforts are being made to improve collection of race and ethnicity data. A major challenge over the course of this pandemic has been collecting data in a standardized format when test results are being reported from a wide variety of laboratories that are affiliated with many different private and governmental entities. We do not believe that the race and ethnicity data are missing at random but also are not able to assess the magnitude of bias that this possibility would introduce, especially given that race and ethnicity appear to be risk factors for infection.

Study strengths include the diversity of OC in terms of socioeconomic and demographic predictors, which provide sufficient power to investigate these factors in our analyses. California was also one of the first states to issue an executive order for residents to stay home, providing data for several months when only essential workers were permitted to work outside the home. Our analyses were able to identify temporal shifts in the demographics of COVID-19 test positivity that likely reflect disparities related to occupation type that are further amplified by household characteristics. Finally, we are able to assess differences in risk for infection and test positivity by comparing our population-level serologic survey to routinely collected (passive) data from county statistics.

The reasons for the spatial, sociodemographic, and economic patterns we discovered are likely complex and broadly related to issues of accessing healthcare and general social determinants of health. The clear disparities in how this disease has manifested in OC point toward the need for approaches that are socio-culturally appropriate and have a focus on health equity. The large amount of missing data and the collection of only broad categories of race and ethnicity information highlight the need for improved data collection. Finally, measures that focus on the hardest-hit communities, including those that involve working with community-based organizations who have experience working with hard-hit demographic and geographic groups to ensure equitable access to health services, may serve as efficient points of intervention for COVID-19.

Appendix Additional information about predictors of test positivity, mortality, and seropositivity during the early coronavirus disease epidemic, Orange County, California, USA.

## References

[1] Dowd JB, Andriano L, Brazel DM, Rotondi V, Block P, Ding X, et al. Demographic science aids in understanding the spread and fatality rates of COVID-19. Proc Natl Acad Sci U S A. 2020;117:9696–8. 10.1073/pnas.2004911117 32300018 PMC7211934

[2] Dorn AV, Cooney RE, Sabin ML. COVID-19 exacerbating inequalities in the US. Lancet. 2020;395:1243–4. 10.1016/S0140-6736(20)30893-X 32305087 PMC7162639

[3] Yancy CW. COVID-19 and African Americans. JAMA. 2020;323:1891–2. 10.1001/jama.2020.6548 32293639

[4] Garg S, Kim L, Whitaker M, O’Halloran A, Cummings C, Holstein R, et al. Hospitalization rates and characteristics of patients hospitalized with laboratory-confirmed coronavirus disease 2019—COVID-NET, 14 states, March 1–30, 2020. MMWR Morb Mortal Wkly Rep. 2020;69:458–64. 10.15585/mmwr.mm6915e3 32298251 PMC7755063

[5] Dondorp AM, Hayat M, Aryal D, Beane A, Schultz MJ. Respiratory support in novel coronavirus disease (COVID-19) patients, with a focus on resource-limited settings. Am J Trop Med Hyg. 2020;102:1191–7. 10.4269/ajtmh.20-0283 32319424 PMC7253105

[6] Bhatraju PK, Ghassemieh BJ, Nichols M, Kim R, Jerome KR, Nalla AK, et al. Covid-19 in critically ill patients in the Seattle region—case series. N Engl J Med. 2020;382:2012–22. 10.1056/NEJMoa2004500 32227758 PMC7143164

[7] Yang X, Yu Y, Xu J, Shu H, Xia J, Liu H, et al. Clinical course and outcomes of critically ill patients with SARS-CoV-2 pneumonia in Wuhan, China: a single-centered, retrospective, observational study. Lancet Respir Med. 2020;8:475–81. 10.1016/S2213-2600(20)30079-5 32105632 PMC7102538

[8] Grasselli G, Pesenti A, Cecconi M. Critical care utilization for the COVID-19 outbreak in Lombardy, Italy: early experience and forecast during an emergency response. JAMA. 2020;323:1545–6. 10.1001/jama.2020.4031 32167538

[9] Maxmen A. Thousands of coronavirus tests are going unused in US labs. Nature. 2020;580:312–3. 10.1038/d41586-020-01068-3 32273619

[10] Orange County Health Care Agency. OC Health Care Agency confirms first case of novel coronavirus. 2020 Jan [cited 2021 Jun 3]. https://mailchi.mp/ochca/novelcoronavirus

[11] World Health Organization. Statement on the second meeting of the International Health Regulations (2005) Emergency Committee regarding the outbreak of novel coronavirus (2019-nCoV) [cited 2021 Jun 3]. https://www.who.int/news/item/30-01-2020-statement-on-the-second-meeting-of-the-international-health-regulations-(2005)-emergency-committee-regarding-the-outbreak-of-novel-coronavirus-(2019-ncov)

[12] US Department of Health and Human Services. Determination that a public health emergency exists. 2020 Jan [cited 2021 Jun 3]. https://www.phe.gov/emergency/news/healthactions/phe/Pages/2019-nCoV.aspx

[13] California Department of Public Health. CDC confirms possible first instance of COVID-19 community transmission in California [cited 2021 Jun 3]. https://www.cdph.ca.gov/Programs/OPA/Pages/NR20-006.aspx

[14] Villasanta A. Coronavirus USA update: Santa Clara County extends local health emergency over nCov. International Business Times. 2020 [cited 2021 Jun 3]. https://www.ibtimes.com/coronavirus-usa-update-santa-clara-county-extends-local-health-emergency-over-ncov-2919607

[15] Sisson P. County declares local health emergency to aid coronavirus response. San Diego Union–Tribune [cited 2021 Jun 3]. https://www.sandiegouniontribune.com/news/health/story/2020-02-14/county-declares-local-health-emergency-to-aid-coronavirus-response

[16] Anderson P. Orange County declares emergency due to coronavirus. NBC Los Angeles [cited 2021 Jun 3]. https://www.nbclosangeles.com/news/local/orange-county-declares-emergency-due-to-coronavirus/2321434

[17] Office of the Mayor of San Francisco. City of San Francisco moves proactively to prepare for possible novel coronavirus activity in the community. 2020 Feb 25 [cited 2021 Jan 19]. https://sfmayor.org/article/city-san-francisco-moves-proactively-prepare-possible-novel-coronavirus-activity-community

[18] Orange County Operational Area Emergency Operations Center. Press release # 010: county issues amended health order and guidance. 2020 Mar 18 [cited 2020 Dec 18]. https://cms.ocgov.com/civicax/filebank/blobdload.aspx?BlobID=114421

[19] State of California. Essential workforce [cited 2020 Dec 18]. https://covid19.ca.gov/essential-workforce

[20] Rho HJ, Brown H. Fremstad S. A basic demographic profile of workers in frontline industries [cited 2021 Jan 19]. https://cepr.net/a-basic-demographic-profile-of-workers-in-frontline-industries

[21] Orange County’s Healthier Together. OC dashboard [cited 2020 Dec 18]. http://www.ochealthiertogether.org/indicators/index/dashboard?alias=ocdashboard&localeId=267&page=2&card=1

[22] Transforming Orange County. Transforming Orange County: assets and needs of Asian Americans and Native Hawaiians and Pacific Islanders [cited 2020 Dec 18]. https://transformingoc.advancingjustice-oc.org

[23] Bruckner TA, Parker DM, Bartell SM, Vieira VM, Khan S, Noymer A, et al. Estimated seroprevalence of SARS-CoV-2 antibodies among adults in Orange County, California. Sci Rep. 2021;11:3081. 10.1038/s41598-021-82662-x 33542329 PMC7862219

[24] Anselin L. Local indicators of spatial association. Geogr Anal. 1995;27:93–115. 10.1111/j.1538-4632.1995.tb00338.x

[25] Zhou G, Afrane YA, Malla S, Githeko AK, Yan G. Active case surveillance, passive case surveillance and asymptomatic malaria parasite screening illustrate different age distribution, spatial clustering and seasonality in western Kenya. Malar J. 2015;14:41. 10.1186/s12936-015-0551-4 25627802 PMC4318448

[26] Mi J, Zhong W, Huang C, Zhang W, Tan L, Ding L. Gender, age and comorbidities as the main prognostic factors in patients with COVID-19 pneumonia. Am J Transl Res. 2020;12:6537–48. 33194050 PMC7653634

[27] Ogedegbe G, Ravenell J, Adhikari S, Butler M, Cook T, Francois F, et al. Assessment of racial/ethnic disparities in hospitalization and mortality in patients with COVID-19 in New York City. JAMA Netw Open. 2020;3:e2026881. 10.1001/jamanetworkopen.2020.26881 33275153 PMC7718605

[28] Rubin-Miller L, Alban C. COVID-19 racial disparities in testing, infection, hospitalization, and death: analysis of epic patient data. 2020 Sep 16 [cited 2021 Jan 8]. https://www.kff.org/coronavirus-covid-19/issue-brief/covid-19-racial-disparities-testing-infection-hospitalization-death-analysis-epic-patient-data

[29] Webb Hooper M, Nápoles AM, Pérez-Stable EJ. COVID-19 and racial/ethnic disparities. JAMA. 2020;323:2466–7. 10.1001/jama.2020.8598 32391864 PMC9310097

[30] Centers for Disease Control and Prevention. Coronavirus disease 2019 (COVID-19). 2020 [cited 2021 Jan 9]. https://www.cdc.gov/coronavirus/2019-ncov/covid-data/investigations-discovery/hospitalization-death-by-race-ethnicity.html

[31] Gover AR, Harper SB, Langton L. Anti-Asian hate crime during the COVID-19 pandemic: exploring the reproduction of inequality. Am J Crim Justice. 2020;45:1–21. 10.1007/s12103-020-09545-1 32837171 PMC7364747

[32] Ng E. The pandemic of hate is giving novel coronavirus disease (COVID-19) a helping hand. Am J Trop Med Hyg. 2020;102:1158–9. 10.4269/ajtmh.20-0285 32314701 PMC7253093

[33] World Health Organization. Social determinants of health [cited 2020 Dec 18]. https://www.who.int/westernpacific/health-topics/social-determinants-of-health

