# Supplementary material for: Predictors of Test Positivity, Mortality, and Seropositivity during the Early Coronavirus Disease Epidemic, Orange County, California, USA
Source: Emerg Infect Dis. 2021 Oct;27(10):2604–18. doi: 10.3201/eid2710.210103 (PMC8462316; doi:10.3201/eid2710.210103)
Supplement: Appendix — Additional information about predictors of test positivity, mortality, and seropositivity during the early coronavirus disease epidemic, Orange County, California, USA. [file 21-0103-Techapp-s1.pdf]

Article DOI: https://doi.org/10.3201/eid2710.210103

Predictors of Test Positivity, Mortality, and Seropositivity during the Early Coronavirus Disease Epidemic, Orange County, California, USA

Appendix

Recruitment for Seroprevalence Survey

The SoapBoxSample database contains email addresses and phone numbers of individuals residing in Orange County. We invited (via email [36.4%] or random-digit telephone dialing [63.6%]) one resident per household to participate, without initial mention of SARS-CoV-2 antibody testing. We did not allow the opportunity to defer the survey to another household member. We recruited up to, but not over, targeted quotas for each sociodemographic stratum, according to the population distribution of each stratum in Orange County. Based on U.S. Census-derived population distributions in OC, we targeted recruitment to ensure adequate sample size of subjects from the following strata: age-by-gender (18 to 34 years, 35 to 54 years, 55 years or above; by male, female), race/ethnicity (Hispanic, Asian non-Hispanic, and other non-Hispanic including white and Black, and household income (<$50,000, $50,000–99,999, and $100,000 or above).

In addition, we encouraged participation of racial/ethnic minorities and/or lower-income adults in several ways. First, for four of the 6 weeks of the survey, we offered the survey in the five most commonly spoken languages in OC (i.e., English, Spanish, Mandarin, Vietnamese, and Korean). Second, we invited all household members of underrepresented groups to receive testing at the drive-thru site. Third, for the last 2 weeks of the survey (given that the quotas for racial/ethnic minorities had not been reached) we targeted particular zip codes with a large proportion of racial/ethnic minorities.

Appendix Table 1. Description of covariates (predictors) used in logistic regressions

Variable	Description	Spatial scale	Transformation
Age	Age of individual	Individual level	Either age groups or 10 y units
Gender	Binary indicator for male or female	Individual level	
Race/ethnicity	Race or ethnicity of individual	Individual level	
% with College Degree	Percentage of adults over age 25 with at least a Bachelor's Degree	Zip code level	Aggregated into quartiles for multivariable regressions
% with Insurance	Percentage of adults over 18 with insurance in the past 5 y	Zip code level	Aggregated into quartiles for multivariable regressions
Population Density	Number of people (in thousands) per square kilometer	Zip code level	Centered on its mean and standardized by standard deviation
House Crowding	Percentage of households with more than 1.0 person per room	Zip code level	
Median Income	Median household income in zip code	Zip code level	Centered on its mean and standardized by standard deviation
% Zip Code SARS-COV-2+	Cumulative incidence of reported COVID-19 cases in zip code, up to August 16th	Zip code level	
COVID ICU patients (std. dev.)	Total number of patients patient with confirmed or suspected COVID-19 in ICU beds (per day)	County level	Centered on its mean and standardized by standard deviation
Time	Continuous variable for time		Centered on its mean and standardized by standard deviation

Appendix Table 2. Logistic regression results for odds ratio of testing sero-positive for SARS-CoV2 in Orange County. This regression differs from Table 4 (https://wwwnc.cdc.gov/EID/article/27/10/21-0103-T4.htm) in that it excludes zip code level covariates other than the cumulative incidence.

	Counts		Adjusted Odds Ratio*
Characteristic	SARS-CoV2+	Total	with (95% CI†)
Age			
18–24	19 (5.43%)	158 (5.35%)	Reference
25–29	31 (8.86%)	234 (7.92%)	1.113 (0.59, 2.08)
30–34	33 (9.43%)	275 (9.31%)	0.98 (0.53, 1.82)
35–39	35 (10%)	328 (11.1%)	0.836 (0.45, 1.54)
40–49	83 (23.71%)	651 (22.04%)	1.066 (0.62, 1.84)
50–59	82 (23.43%)	659 (22.31%)	1.071 (0.62, 1.86)
60–69	46 (13.14%)	418 (14.15%)	0.99 (0.55, 1.8)
70–79	18 (5.14%)	188 (6.36%)	0.931 (0.46, 1.9)
80+	3 (0.86%)	43 (1.46%)	0.676 (0.19, 2.42)
Sex			
Female	222 (63.43%)	1668 (56.47%)	Reference
Male	128 (36.57%)	1286 (43.53%)	0.731 (0.58, 0.92)
Race‡			
White	108 (30.86%)	1228 (41.57%)	Reference
Asian	47 (13.43%)	435 (14.73%)	1.286 (0.89, 1.87)
Black	5 (1.43%)	42 (1.42%)	1.309 (0.5, 3.43)
Hispanic	162 (46.29%)	1010 (34.19%)	1.578 (1.2, 2.08)
Pacific Islander	3 (0.86%)	12 (0.41%)	3.62 (1, 13.16)
Unknown	25 (7.14%)	227 (7.68%)	1.254 (0.78, 2.01)
% Zip Code SARS-CoV2+§			1.473 (1.24, 1.74)

*Model intercept represents odds of testing sero-positive for SARS-CoV2 for a white female diagnosed with SARS-CoV2 in the 18–24 age group in a zip code in the first quartile of college degree and insured with the average population density, and average percent of SARS-CoV2 positive individuals in Orange County. The odds of this individual testing sero-positive is estimated to be 0.074 (0.031,0.178)

†95% confidence interval computed with robust standard errors

‡Native American/Native Alaskan race group not included in analysis due to lack of data, no individual of this race group tested seropositive

§Number of individuals who tested positive in individual's zip code reported to OC Public Health Department from March 1st to August 16th, divided by estimated population of zip code

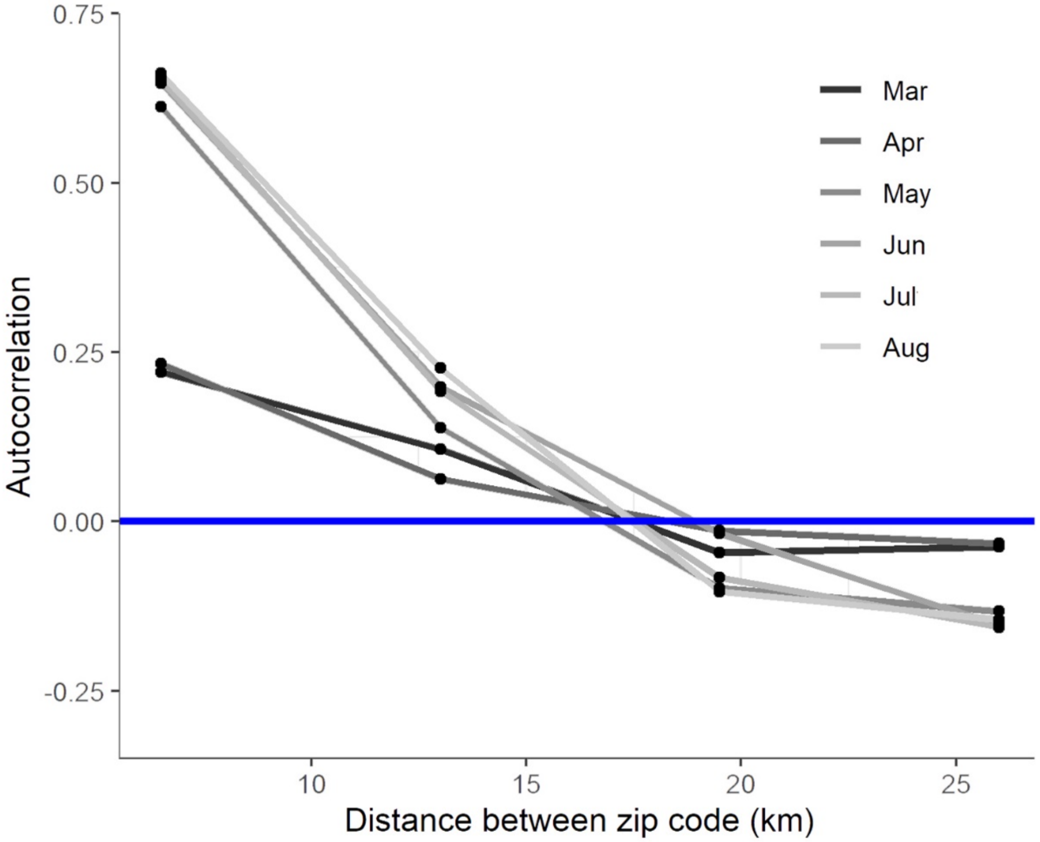

Appendix Figure 1. Spatial correlogram indicating degree of clustering (along y-axis) in reported COVID-19 case incidence by distance (km) away from neighboring zip codes in Orange County, California. Clustering in March is indicated by the black line and subsequent months are indicated by progressively lighter colors. Clustering has been present since the first month (March) but since May the pattern has been stronger and has remained consistent.

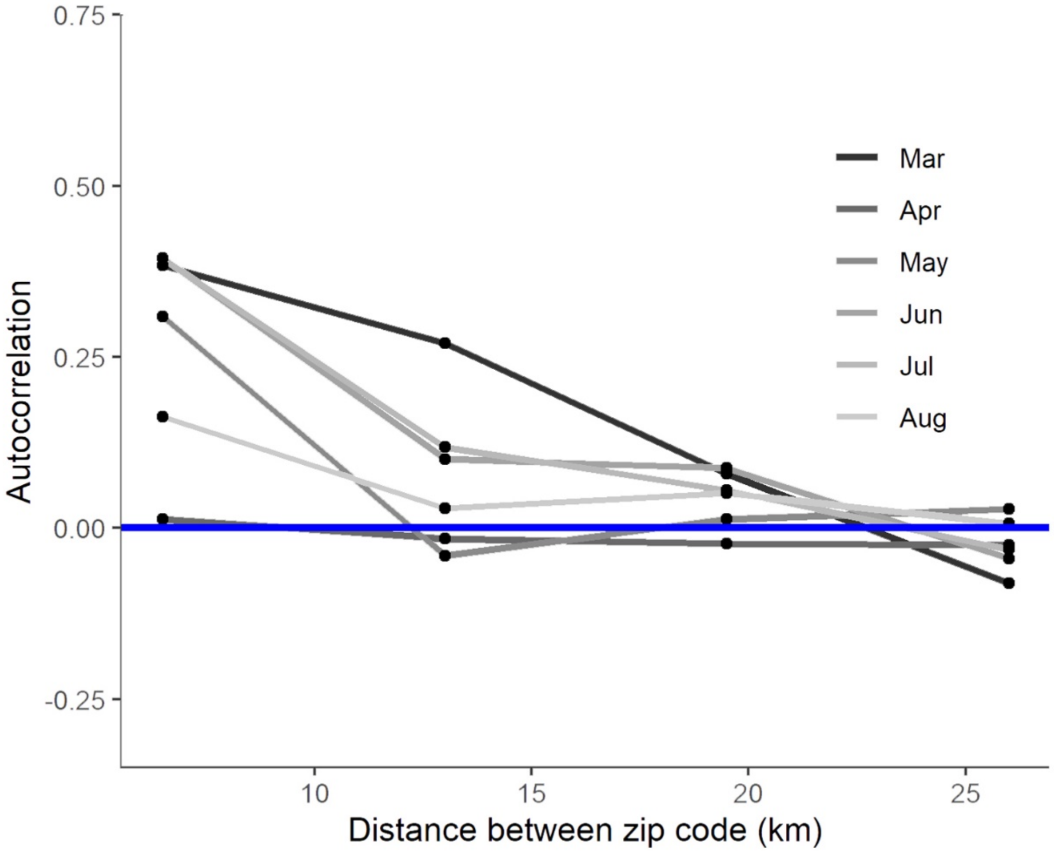

Appendix Figure 2. Spatial correlogram indicating degree of clustering (along y-axis) in COVID-19 testing intensity by distance (km) away from neighboring zip codes in Orange County, California. Clustering in March is indicated by the black line and subsequent months are indicated by progressively lighter colors. Clustering in March was strong and by May was almost non-existent at any spatial extent (May's correlogram falls along the horizontal blue line, with an autocorrelation coefficient near zero). Clustering in testing intensity was again strong in June and July.

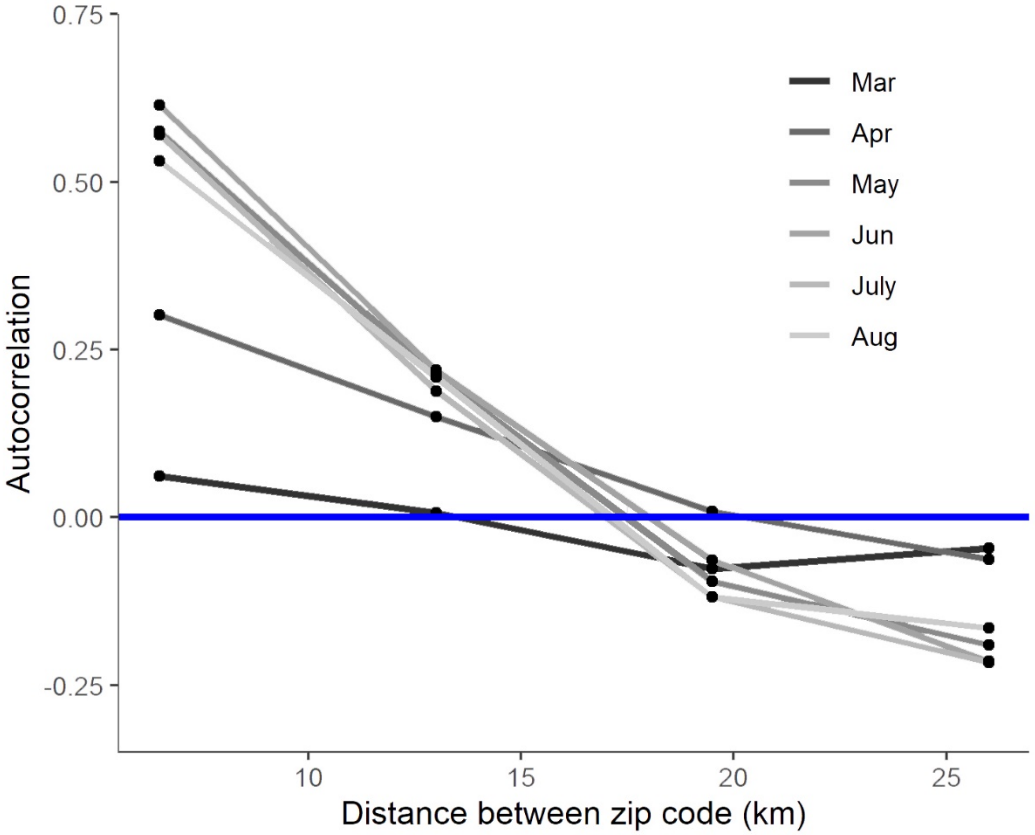

Appendix Figure 3. Spatial correlogram indicating degree of clustering (along y-axis) in COVID-19 test positivity by distance (km) away from neighboring zip codes in Orange County. There was almost no clustering in test positivity in March, at the beginning of the epidemic (the line for March falls near the zero point). Clustering in test positivity was apparent in April, increased in May, and has remained at approximately the same level since.
